# Modulators of the Human Voltage-Gated Proton Channel H_v_1

**DOI:** 10.3390/ph18101480

**Published:** 2025-10-01

**Authors:** Jesús Borrego, Beáta Mészáros, Tibor G. Szanto, Russo Teklu Teshome, Éva Korpos, Zoltan Varga, Ferenc Papp

**Affiliations:** 1 Department of Biophysics and Cell Biology, Faculty of Medicine, University of Debrecen, Egyetem ter 1, H-4032 Debrecen, Hungarymeszaros.beata@med.unideb.hu (B.M.);; 2 MTA-DE Cell Biology and Signalling Research Group, Faculty of Medicine, University of Debrecen, Egyetem ter 1, H-4032 Debrecen, Hungary

**Keywords:** peptide inhibitors, small molecule inhibitors, H_v_1 activators, human H_v_1

## Abstract

The voltage-gated proton channel (H_v_1) selectively transports protons (H^+^) across biological membranes in response to membrane potential changes. H_v_1 is assembled as a dimer, and unlike most voltage-gated ion channels, it lacks a traditional central pore domain; instead, the voltage-sensing domain (VSD) of each monomer facilitates proton conduction via a hydrogen-bond network. H_v_1 is widely expressed in various human cell types (e.g., immune cells, sperm, etc.) including tumor cells. In tumor cells, the accumulation of acidic intermediates generated by glycolysis under hypoxic conditions or ROS production leads to significant cytosolic acidification. H_v_1 can remove protons from the cytosol rapidly, contributing to the adaptation of the cells to the tumor microenvironment, which may have significant consequences in tumor cell survival, proliferation, and progression. Therefore, H_v_1 may be very promising not only as a tumor marker but also as a potential therapeutic target in oncology. Molecules that modulate the proton flux through H_v_1 can be divided into two broad groups: inhibitors and activators. H_v_1 inhibitors can be simple ions, small molecules, lipids, and peptides. In contrast, fewer H_v_1 activators are known, including albumin, NH29, quercetin, and arachidonic acid. The mechanism of action of some inhibitors is well described, but not all. H_v_1 modulation has profound effects on cellular physiology, especially under stress or pathological conditions, like cancer and inflammation. The therapeutic application of selective H_v_1 inhibitors or activators could be a very promising strategy in the treatment of several serious diseases.

## 1. Introduction

### 1.1. Voltage-Gated Proton Channels

Voltage-gated proton channels (H_vs_) represent a unique family of voltage-gated ion channels responsible for the selective passage of protons (H^+^) across biological membranes in response to changes in membrane potential. The first direct electrophysiological evidence of voltage-gated and pH-sensitive proton currents was published in the early 1980s [1]. This pioneering study laid the groundwork for understanding voltage-gated proton channels, whose molecule was later identified in 2006 [2,3]. The voltage-sensing domain (VSD) of H_v_ channels serves the dual function of sensing voltage changes and facilitating proton permeation [4]. Recently, our understanding of the voltage-gated proton channel family has been expanded with the discovery and characterization of three new proton channel members—AcH_v_1, AcH_v_2, and AcH_v_3—identified in the mollusk “*Aplysia californica*” [5]. More recently, another H_v_ channel was announced, namely H_v_4, which was identified only in bivalvian mollusks [6].

H_v_1 shows a wide tissue distribution pattern and presents diverse functions. It is expressed by several cell types in physiological conditions, like different immune cell types (neutrophils [7], eosinophils [8], macrophages [9], microglia [10], T and B cells [11,12]), human airway epithelium cells [13], human cardiac fibroblasts [14], chorion-derived mesenchymal stem cells [15], pancreatic islet β-cells [16], sperm [17], and oocytes [18]. H_v_1 is also expressed in pathological situations, such as by tumor cells: malignant B cells [19], glioblastoma multiforme cells [20], leukemic Jurkat T cells [21], breast cancer cells [22,23], and colorectal cancer cell lines [24].

### 1.2. Structure of H_v_1

**Figure 1:**
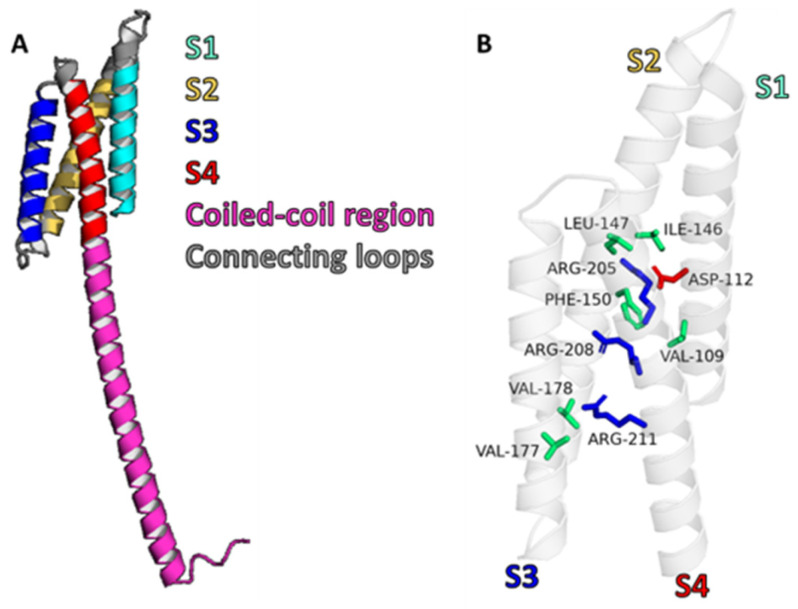
Structural features of the human H_v_1 channel. (**A**) Cartoon representation of truncated hH_v_1 lacking the N-terminus. Transmembrane helices S1, S2, S3, and S4 are shown in cyan, yellow, blue, and red, respectively. The intracellular C-terminal coiled-coil region is depicted in pink, and connecting loops are shown in gray. (**B**) Transmembrane helices are displayed as a semi-transparent white cartoon. Residues forming the hydrophobic plug are highlighted as green sticks, the selectivity filter as red sticks, and voltage-sensing arginines as blue sticks. The monomeric AlphaFold-predicted structure AF-Q96D96-F1 was used for figure generation.

Structurally, Hv channels differ from classic voltage-gated ion channels (VGICs), such as sodium (Nav), potassium (Kv), or calcium (Cav) ion channels. Typically, VGICs are composed of either homo- or heterotetrameric structures (Kv) or a single polypeptide chain of four homologous domains (Nav and Cav); each of the four subunits or domains consists of six transmembrane α-helical segments linked by intra- and extracellular loops. Of these, segments S1–S4 form the voltage-sensing domain (VSD) that regulates channel opening upon membrane depolarization, while segments S5 and S6 and the connecting extracellular pore loops (P-loops) constitute the pore domain. However, Hv channels have a unique architecture, as they are homodimers, composed of only two identical subunits, and both subunits contain four transmembrane segments (S1–S4) serving as VSDs (Figure 1). Unlike most VGICs, there is no distinct pore structure in H_v_1 channels. Instead, protons permeate through each VSD individually, each with its intrinsic conduction pathway [25,26,27]. However, when monomers assemble into dimer proton channels in the membrane, cooperativity can be observed between them, and the dimer channel only becomes conductive when both monomers are activated [28,29,30]. More specifically, proton conduction in H_v_1 channels may be described by the Grotthus mechanism, that is, H^+^ ions hop along a robust water wire within the channel structure, facilitated by specific water–protein interactions [31]. However, several studies have shown that H_v_1 contains a dry region. This region disrupts the water wire such that protonation of one or more amino acids may occur, allowing protons to hop across via a hydrogen-bond chain mechanism [32,33,34]. The S4 segment terminates with a coiled-coil region facing the cell interior (Figure 1A), which contributes to the dimerization of the channel [25,26,35,36]. The voltage-gated proton channels are highly selective for H^+^ ions, ensured by specific charged residues—the open H_v_1 channel requires an aspartate at 112 (Asp112) in the S1 segment and an arginine in the S4 helix, forming a narrow region that conducts protons selectively [37] (Figure 1B). Some studies propose that Asp112 interacts with Arg211 (R3) [38,39], while others propose its interaction with Arg208 (R2). Specifically, truncation of the S4 segment between R2 and R3 [27] or mutation of R3 to alanine [40] did not alter the proton selectivity of H_v_1. The voltage-sensing part of the channel contains three positively charged arginine residues, known as gating arginines (Arg205 (R1), Arg208 (R2), and Arg211 (R3)); all are located in the S4 domain (Figure 1). These gating arginines interact with their negatively charged countercharge residues in the S1–S3 domains and “detect” the change in the membrane potential [41]. In the case of the Ci-H_v_1 ion channel, attempts were made to isolate and measure the gating charge [42,43]. When the cell membrane is depolarized, or when there is an elevated proton concentration in the cyctosol or the contrary, the extracellular pH is alkaline, and channel activation is triggered [30]. In H_v_1 channels, there is no inactivation mechanism; only open or closed states are observed [44]. In response to membrane depolarization, the gating arginines are repelled toward the extracellular space. These changes are mediated by two steps, as described by the three-state model, based on H_v_1 current and fluorescence recordings [45,46,47]. According to this model, the structural changes between the different states result in a notable shift in the salt-bridge interaction network formed between S3 and S4. It should also be mentioned that there are articles that assume less or more than three states [48,49]. Channel activation critically influences both water distribution and proton permeability. In the open state, internal water molecules interact with charged residues through hydrogen bonds, often forming a continuous network that can conduct protons from the cytosol to the extracellular space [31]. However, multiple studies indicate the presence of intermittent dry regions within the pore that disrupt the continuity of this water wire. In such cases, proton transport may require transient protonation of amino acid side chains, allowing protons to “hop” across the gap via a hydrogen-bond chain mechanism. This dual mode of conduction highlights the dynamic interplay between water molecules and protein residues in regulating H_v_1 channel function [32,33,34]. In addition to voltage gating, the H_v_1 channel can be regulated by relative pH changes. Acidification of the cell interior alters the voltage gating. The response of H_v_1 proton conductance to changes in pH gradient is approximately 40 mV/ΔpH [47,48,50,51]. This, together with its high proton selectivity, is perhaps the most characteristic feature of the channel. The pH dependence of gating is fundamental to the role of H_v_1 in maintaining cellular pH homeostasis. This aspect is critical because it forms the basis for understanding the potential role of the channel in cancer cell survival.

### 1.3. Functions of H_v_1 and Its Role in Cancer

**Figure 2:**
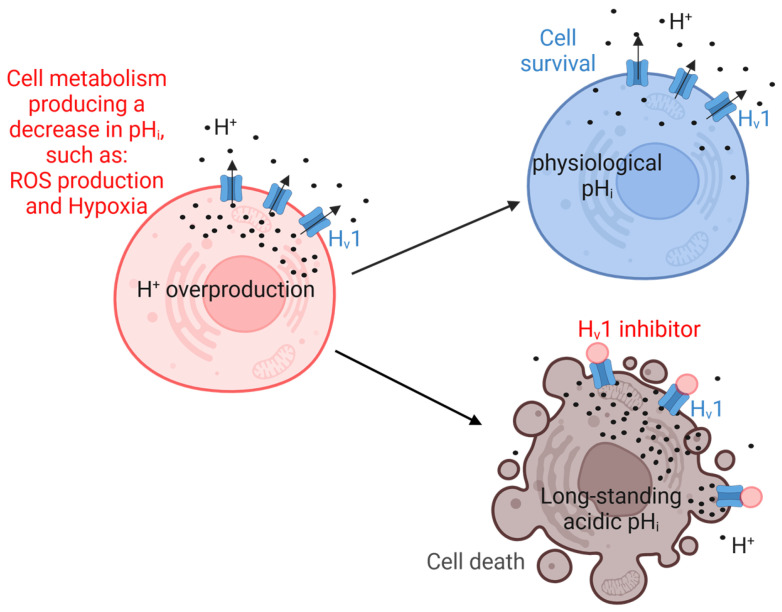
Schematic representation of how H_v_1 inhibition is responsible for promoting cell death by preventing proton extrusion. (**top**) The normal mechanism for sustained ROS production or hypoxia elevates intracellular H+, and the action of H_v_1 compensates for the accumulation of H+. (**bottom**) When H_v_1 is inhibited, one consequence is that protons accumulate, resulting in a decrease in pH_i_. This inhibits the action of NOX2, which in turn reduces the production of ROS, maintaining the intracellular acidification that promotes cell death. Created in https://BioRender.com, accessed on 12 September 2025.

Studies have revealed the diverse roles of H_v_1 in cancer cell types, particularly the ability to rapidly and robustly transport protons from the cytosol, thus regulating the intracellular pH (pH_i_) of cells. Therefore, H_v_1 is involved in many processes that can lead to a decrease in pH_i_, such as the NADPH oxidase-dependent production of ROS by immune cells [52] or the accumulation of acidic intermediates during glycolysis in tumor cells under hypoxic conditions [53]. Inhibition of H_v_1 results in at least two things: depolarization of the cell membrane (in a few milliseconds) [54], and a decrease in pH_i_ (on a timescale of seconds to minutes) [55]. However, in many cases the role of H_v_1 in different cellular processes has not yet been clarified. Without being exhaustive, the role of H_v_1 has been highlighted in cancer cell migration and proliferation, cell survival and apoptosis [10,21], sustained calcium entry [56], neutrophil migration and superoxide production [56], sperm capacitation and motility [17], participation in optimal B-cell receptor signaling and redox control in human B lymphocytes [19], and regulation of insulin secretion [16]. Moreover, H_v_1 plays a crucial role in cancer development, progression, and metastasis formation, allowing H_v_1 to become a potential target in tumor therapy [23]. During processes producing ROS or under hypoxic conditions, tumor cells produce elevated proton concentrations in the cytosol. In the hypoxic tumor microenvironment, tumor cells have high glycolytic activity, converting glucose to acidic metabolites [57]. When pH_i_ reaches a critical value relative to the extracellular pH, the threshold potential for the channel opening of H_v_1 shifts sufficiently towards a more negative membrane potential, releasing protons from the cell, thereby reducing the proton concentration in the cytosol. Proton extrusion consequently affects the extracellular proton concentration, which may contribute to maintaining the acidic tumor microenvironment [57,58]. If, however, the H_v_1 channel is inhibited, pH_i_ in tumor cells is expected to remain permanently low, promoting cell death (Figure 2). Furthermore, since H_v_1 may contribute to the acidification of the extracellular milieu, which suppresses antitumoral T-cell responses, proton extrusion from the IC to the EC also promotes tumor growth and progression by inhibiting the immune system [57,58,59]. H_v_1 may also be important in the normal function and regulation of the nervous system, and accordingly, H_v_1 can be responsible for various neurological diseases [60]. Thus, finding or developing a suitable H_v_1 inhibitor or activator has been intensively pursued. Functional studies have explored a number of known H_v_1 inhibitors or activators, but for most of these, selectivity (whether they affect other ion channels) has not been investigated. H_v_1 inhibition by Zn^2+^ or ClGBI produced a significant acidification of Jurkat cells and induced cell death by apoptosis [21]. ClGBI also decreased the cell viability of tumorigenic breast cells along with a decrease in pH_i_ [22]. Inhibiting H_v_1 with Zn^2+^ significantly reduced pH_i_, decreasing cell survival and migration of a glioblastoma multiforme cell line [20]. Moreover, Zn^2+^ markedly decreased the cell invasion and migration of a colorectal cell line (SW620, HT29; [24]). Zn^2+^ ions also induced apoptosis in human highly metastatic glioma and effectively suppressed cancer growth and metastasis by reducing proton extrusion and downregulating gelatinase activity [61]. Myeloid-derived suppressor cells (MDSCs) also express H_v_1 [62]. ClGBI significantly decreased the migration and osteogenic differentiation of chorion-derived mesenchymal stem cells [15]. Corza6 also blocked the acrosome reaction during capacitation of sperm and inhibited ROS production in human white blood cells (WBCs) [63]. Inhibition of neuronal H_v_1 by a newly discovered inhibitor, YHV98-4, reduced intracellular alkalization and ROS production in peripheral sensory neurons [64]. Interestingly, macrophages are an important source of arachidonic acid metabolites, which are able to activate H_v_1 [65].

Since the inhibitors are not selective for H_v_1, it is difficult to estimate to what extent these effects are due to the inhibition of proton currents or due to other reasons. Recently, it has been shown that ClGBI is not a specific inhibitor of H_v_1 since it inhibits several other channels [66] on lymphocytes. 2GBI, the precursor of ClGBI, has been shown to bind to NLRP3, which leads to inflammasome assembly and activation. This function of 2GBI is independent of H_v_1 since the impact on inflammasome is also detected in bone marrow-derived macrophages where the *HVCN1* gene is knocked down [67]. Therefore, the use of H_v_1 knockout (KO) mice or the silencing *HVCN1* gene is needed in additional pharmacological studies to understand and explore the function of H_v_1 in health and disease [23,24,62,68,69]. Targeting the H_v_1 proton channel in biological systems is pretty challenging due to its broad tissue distribution pattern. While considering H_v_1 as a potential target in cancer therapy, it has to be taken into consideration that H_v_1 is also expressed by immune cells that present anti-cancer properties, such as cytotoxic T cells and B cells. However, it is important to note that there are two known isoforms of H_v_1; the shorter isoform (H_v_1s) is shorter by 20 amino acids at the N-terminal compared to the long isoform. The appearance of H_v_1s may have prognostic and therapeutic significance, as the appearance of the shorter isoform may contribute to tumor progression and proliferation, as presented in studies on the MDA-MB-231 breast cancer cell line and malignant B cells [19,22]. The development of H_v_1-dependent tumor therapy raises further questions and possibilities, as more than 100 somatic mutations in H_v_1 have been described in numerous tumor types (see the COSMIC and ClinVar databases). Without claiming to be exhaustive, these include the following cancer types: Burkitt lymphoma; glioblastoma multiforme; breast ductal carcinoma; colon, lung, and prostate adenocarcinoma; malignant melanoma; etc. The mutations in the S4 segments of H_v_1 resulted in different biophysical properties in channel function [70]. The consequences of the mutations in tumor proliferation and progression are still unknown. Thus, while pharmacological tools and KO models are essential, a deeper understanding of H_v_1 function will require explicit, testable mechanistic models, analogous to those developed for the respiratory burst in phagocytes, where quantitative evaluation of proton currents, channel kinetics, and oxidase-derived electron fluxes have provided robust functional insights.

## 2. H_v_1 Modulators

The proton current through the H_v_1 channel can be modulated by a diverse array of molecules at concentrations ranging from nanomolar (nM) to micromolar (µM) levels (Table 1). These H_v_1 modulators are simple ions (e.g., Zn^2+^), small molecules (e.g., HIF, ClGBI), unsaturated fatty acids (e.g., arachidonic acid), and peptides (e.g., HaTx, GsAF-I). Based on their effect on proton currents, H_v_1 modulators can be divided into two groups: inhibitors and activators.

### 2.1. H_v_1 Inhibitors

**Figure 3:**
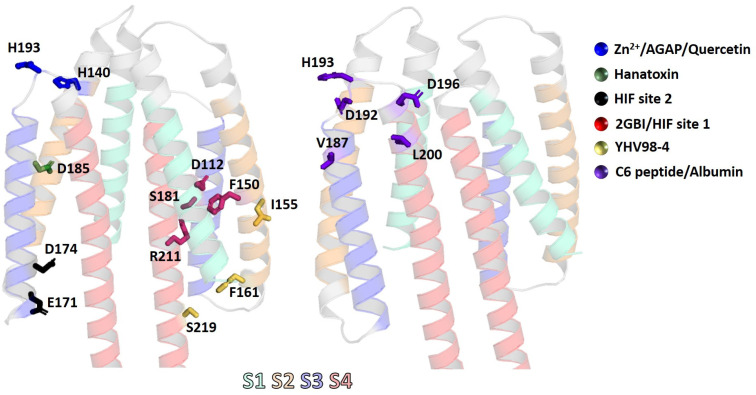
Binding sites of H_v_1 modulators. The intermembrane region of the human H_v_1 channel and its respective loops. The monomeric AlphaFold-predicted structure AF-Q96D96-F1 was used to generate the hH_v_1 dimer. Transmembrane helices S1, S2, S3, and S4 are shown as transparent cartoons, while residues that are involved in the binding of different H_v_1 modulators are illustrated in stick representations.

One of the earliest identified inhibitors of H_v_1 was Zn^2+^ [91]. This divalent cation inhibits H_v_1 in a reversible manner by binding to the closed conformation of H_v_1, thereby reducing the open probability of the channel and stabilizing its non-conducting state [92]. The binding site of Zn^2+^ is in the S3–S4 loop, with H140 and H193 playing key roles in binding (Figure 3) [3,93]. Interestingly, dimerization of the hH_v_1 channel creates novel binding sites for divalent cations by reorienting and juxtaposing key coordinating residues that remain spatially separated in the monomeric state. In dimeric assembly, histidines and acidic side chains contributed by adjacent protomers are positioned in close proximity, establishing unique coordination geometries that are absent in the monomeric channel [93,94]. In addition to Zn^2+^, several other inhibitors exhibit a similar mechanism of action. For instance, the mutated version of AGAP-W38F (anti-tumor analgesic peptide), isolated from the scorpion *Buthus martensii*, behaves as an H_v_1 inhibitor by trapping the S4 voltage sensor in its deactivated state [76]. In contrast, while Zn^2+^ inhibition demonstrates a high degree of pH dependency, AGAP exhibits a reduced sensitivity to pH changes. Intriguingly, the binding pocket of AGAP-W38F partially overlaps with that of Zn^2+^, sharing critical residues H140 and H193 [3,76]. Other molecules that also stabilize the channel in its closed conformation are cholesterol [88], Oxophench [72], PNX61442 [86], a molecule called 13 [80], and NH17 [85]; however, the molecular determinants underlying their interaction with H_v_1 are still poorly understood. For example, cholesterol does not directly interact with hH_v_1 residues but instead inhibits channel activity indirectly by altering the conformational kinetics of the voltage-sensing S4 domain and modifying the biophysical properties of the surrounding membrane [88,95]. In fact, a recent study concluded that cholesterol probably does not affect H_v_1 directly, but indirectly. The data obtained can be explained more plausibly by the fact that H_v_1 preferentially associates with cholesterol-dependent lipid domains, or “rafts” [96].

Molecules derived from guanidine have also been identified as H_v_1 inhibitors [83]. 2-guanidinobenzimidazole (2GBI) has been observed to bind to the voltage-sensing domain (VSD) when the channel is in its open conformation (Figure 3). The binding pocket, accessible from the intracellular side of the membrane, involves amino acids D112, F150, S181, and R211, with F150 being critical for the interaction [83,97]. However, due to its high polarity, 2GBI has low permeability through cell membranes, thereby limiting its usage in pharmacological studies and precluding it from being a drug candidate [84,98]. To address this limitation, a derivative called Cl-guanidinobenzimidazole (ClGBI) was developed, which exhibits enhanced membrane permeability, enabling access to the intracellular domain of the channel and blocking it with higher binding affinity [83]. However, ClGBI has been shown to inhibit not only H_v_1 at micromolar concentrations but also other ion channels, which greatly limits its future use as a tool in functional studies or as a potential drug candidate [66].

Another small molecule derivative, HIF (3-(2-amino-5-methyl-1H-imidazol-4-yl)-1-(3,5-difluorophenyl)propan-1-one), exhibits dual mechanisms of action depending on its interaction site within the channel. When HIF binds to “site 1”, its binding mechanism is similar to that of 2GBI, and it was confirmed that mutations of D112 and F150 abolish the inhibitory effect of HIF against H_v_1. In contrast, binding to “site 2”, involving residues E171 and D174, leads to a slower recovery from inhibition [82]. Located near the 2GBI pocket, another binding pocket has been identified that accommodates the small molecule modulator of YHV98-4. This pocket is formed by amino acids I155, F161, and S219 (Figure 3). Molecular dynamic (MD) simulations suggest that upon binding to H_v_1, YHV98-4 inhibits proton conduction by disrupting the water wires necessary for proton transfer [64]. It is important to note that in the article, the concepts of IC_50_ and K_d_ are confused, mostly because the inhibition does not saturate around zero but rather around 0.5. Therefore, IC_50_ should not be used, but rather K_d_.

Certain antidepressant drugs, e.g., imipramine [74], antitussive drugs, e.g., dextromethorphan [87], and antipsychotic drugs, such as chlorpromazine, haloperidol, and clozapine [75,81], have been shown to inhibit the voltage-gated proton currents in BV2 microglial cells. These drugs penetrate the cell membrane in their uncharged, neutral form and subsequently undergo protonation in the cytosol. The charged forms of the drugs then block the proton channel intracellularly. Since these molecules are protonated, they may reduce the pH gradient, potentially leading to the reduction of the proton current. However, no changes in the reversal potential of the current were observed, suggesting that the inhibition mechanism does not directly alter the electrochemical equilibrium. Moreover, a similar inhibitory effect has been reported for other molecules, such as epigallocatechin-3-gallate (EGCG), the principal bioactive constituent of green tea [78]. Further studies are needed to fully explore the mechanism by which these protonated molecules inhibit proton currents.

Besides the peptide inhibitor AGAP-W38F, other peptides have also been reported as H_v_1 inhibitors. One of these is the synthetic C6 peptide, which binds with nanomolar affinity to both the S3 and S4 loops of the dimer hH_v_1 in a cooperative manner. This cooperative binding causes the C6-bound channels to activate at a more positive membrane potential, i.e., C6 slows the activation of H_v_1. The most critical residues for this interaction are V187, E192, H193, E196, and L200 (Figure 3) [71]. The venom of the tarantula *Grammostola rosea* has also been identified as the source of three H_v_1 inhibitors. The first of these is Hanatoxin (HaTx), one of the earliest reported peptides that is capable of inhibiting H_v_1. Extracellular application of HaTx produced inhibition of H_v_1 proton currents, shifting the activation of the channel to more positive voltages [73]. Based on its interaction with Kv1.2, HaTx is assumed to partition into the membrane before interacting with a binding site at the protein–lipid interface. D185 plays a key role in this interaction, as shown by site-directed mutagenesis (D185A), which effectively abolishes the inhibitory effect of HaTx [73]. The other two peptides isolated from *Grammostola rosea*, Gr1b (GsAF-I) and Gr2c (GsAF-II), showed similar effects to HaTx; they shifted the activation threshold potential of H_v_1 towards more positive potentials, reduced the H_v_1 current in a membrane potential-dependent manner, and stabilized the channel in its closed state ([77]). However, the molecular determinants responsible for these inhibitory effects are still unknown.

It should also be noted that several of the substances listed in Table 1 can be considered weak base compounds (Fluoxetine, Chlorpromazine, Imipramine, Haloperidol, Clozapine, Desipramine, Dextromethorphan, Olanzapine). After passing through the membrane, these molecules can bind to protons inside the cell, thereby reducing the pHi. This may indicate apparent inhibition of the H_v_1 channel, as the increased pHi affects the function of H_v_1, reducing its probability of opening [99].

### 2.2. H_v_1 Activators

**Figure 4:**
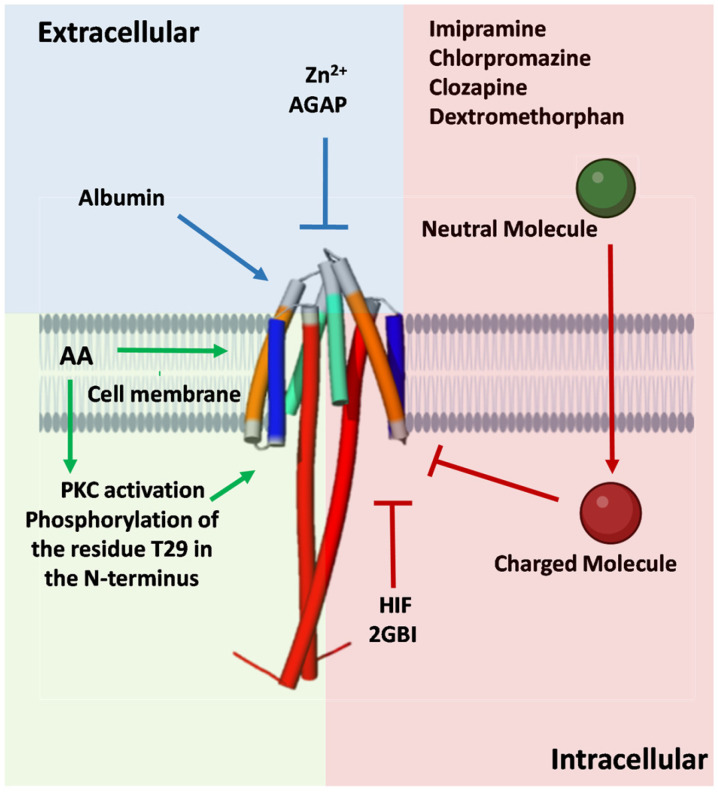
Different modulation mechanisms of the H_v_1 channel. The monomeric AlphaFold-predicted structure (AF-Q96D96-F1) was used to generate the hH_v_1 dimer embedded in a cell membrane. H_v_1 channel modulators influence proton currents through direct or indirect mechanisms acting extracellularly, intracellularly, or within the membrane. Pointed arrows indicate activation pathways (blue and green), whereas flat-headed arrows indicate inhibition (red). AA, arachidonic acid.

In contrast to the H_v_1 inhibitors, only a few H_v_1 activators have been described so far. One notable example is albumin (Alb), which has been shown to enhance the opening probability and increase proton currents in H_v_1. A single Alb molecule binds to the dimeric hH_v_1 channel at both voltage-sensor domains (VSDs), specifically to the external S3-S4 loops. Mutations H193C and L200C in H_v_1 fully eliminated Alb activation, suggesting that these two residues mediate direct interaction with Alb [89]. Interestingly, these residues are also involved in the inhibitory effects of Zn^2+^ and the C6 peptide. Similar to Alb, NH29 stabilizes the H_v_1 channel in its open state [85]. External application of NH29 increased proton currents at all test potentials, primarily due to a significant hyperpolarizing shift in the conductance–voltage relationship [85]. In some cases, activators can enhance H^+^ currents through indirect mechanisms. Onion peel extract (OPE), from *Allium cepa* L., was found to modulate H_v_1 channel opening and activates the channel at more negative membrane voltages. Subsequent studies revealed that quercetin, the major active component in OPE, is responsible for this effect. The activation of H_v_1 induced by OPE was inhibited by 10 µM Zn^2+^ and GF109203X (GFX), a specific protein kinase C (PKC) inhibitor. It was concluded that the pro-oxidant effects of quercetin play a significant role in OPE-induced activation of H_v_1 as well as its probable involvement in PKC signaling pathways [90]. While the precise interaction between OPE, PKC, and H_v_1 remains unclear, similar PKC-related mechanisms have been observed in H_v_1 activation by lipopolysaccharides (LPSs). The acute addition of LPSs increased the H_v_1 channel activity that was abolished by GFX. However, the activating effect of LPSs on H_v_1 disappeared after 24 h incubation with LPSs, instead acting as an inhibitor compound over time [100]. Arachidonic acid (AA) also activates H_v_1 channels, enhancing the proton current both by direct interaction and by activating PKC [101]. The phenomenon of phosphorylation is consistent with prior observations of OPE and lipopolysaccharides. However, in the truncated isoform of mH_v_1, which lacks the N-terminal region that contains the phosphorylation site T29, AA addition still enhanced H_v_1 currents. This indicates that AA can act directly on mH_v_1, with channel modulation occurring after AA incorporates into the membrane rather than through interaction from the extracellular side. The hydrophilic head group of AA is essential for this effect, whereas the molecular charge does not appear to play a critical role [65]. Figure 3 highlights the principal binding sites for H_v_1 channel activators and inhibitors, whereas Figure 4 illustrates representative mechanisms by which these modulators influence channel activity.

## 3. Conclusions

H_v_1 has emerged as a pivotal player in numerous physiological and pathological contexts, particularly in cancer and inflammation. The growing repertoire of H_v_1 modulators not only deepens our understanding of proton channel function but also opens the door to promising therapeutic strategies. Targeting H_v_1 with selective modulators may soon translate into meaningful advances in disease treatment.

## Figures and Tables

**Table 1:** IC_50_ of different H_v_1 modulators.

Compound	Type	IC_50_	Effect on the Channel	Reference
Peptide C6	Peptide	31 nM	Inhibitor (ec)	[71]
Oxopench	Small molecule	819 nM	Inhibitor	[72]
Zn^2+^	Cation	1.9 µM	Inhibitor (ec)	[3]
Hanatoxin	Peptide	2 µM	Inhibitor (memb)	[73]
Fluoxetine	Small molecule	2.1 µM	Inhibitor (ic)	[74]
Chlorpromazine	Small molecule	2.2 µM	Inhibitor (ic)	[75]
AGAP/W38F	Peptide	2.5 µM	Inhibitor (ec)	[76]
Gr1b/GsAF-l	Peptide	3.2 µM	Inhibitor (ec)	[77]
Gr2c/GsAF-ll	Peptide	3.6 µM	Inhibitor (ec)	[77]
Epigallocatechin	Small molecule	3.7 µM	Inhibitor (ic)	[78]
Cd^2+^	Cation	5 µM	Inhibitor (ec)	[79]
Imipramine	Small molecule	5.7 µM	Inhibitor (ic)	[74]
Mitriptyline	Small molecule	5.8 µM	Inhibitor (ic)	[74]
Haloperidol	Small molecule	8.4 µM	Inhibitor (ic)	[75]
13	Small molecule	8.5 µM	Inhibitor (ic)	[80]
Clozapine	Small molecule	9.8 µM	Inhibitor (ic)	[81]
Desipramine	Small molecule	<10 µM	Inhibitor (ic)	[74]
HIF	Small molecule	26 µM	Inhibitor (ic)	[82]
ClGBI	Small molecule	26.3 µM	Inhibitor (ic)	[83]
2GBI	Small molecule	38 µM	Inhibitor (ic)	[84]
NH17	Small molecule	>50 µM	Inhibitor (ic)	[85]
PNX52429	Small molecule	>50 µM	Inhibitor	[86]
PNX61442	Small molecule	50 µM	Inhibitor	[86]
Dextromethorphan	Small molecule	51.7 µM	Inhibitor (ic)	[87]
Olanzapine	Small molecule	84 µM	Inhibitor (ic)	[81]
Cholesterol	Lipid	~10% (wt/wt, to total membrane lipids)	Inhibitor (memb)	[88]
Albumin	Protein	158 µM	Activator (ec)	[89]
Arachidonic acid	Lipid	10-100 µM	Activator (memb)	[65]
NH29	Small molecule	50 µM	Activator	[85]
OPE (onion peel extract)	Organic extract	30 µg/ml	Activator (ec)	[90]

## Data Availability

No new data were created or analyzed in this study. Data sharing is not applicable to this article.

## Abbreviations

The following abbreviations are used in this manuscript:

VSD	Voltage-sensing domain
ROS	Reactive oxygen species
VGIC	Voltage-gated ion channel
ic	Intracellular
ec	Extracellular
memb	Membrane
NADPH	Nicotinamide adenine dinucleotide phosphate
SW620	A colorectal cell line
HT29	A colorectal cell line
MDSC	Myeloid-derived suppressor cell
WBC	White blood cell
NLRP3	NLR family pyrin domain-containing 3
KO	Knockout
HVCN1	The gene that encodes “Voltage-gated hydrogen channel 1”
NOX2	NADPH oxidase 2
HIF	3-(2-amino-5-methyl-1H-imidazol-4-yl)-1-(3,5-difluorophenyl)propan-1-one
AGAP	Anti-tumor analgesic peptide, isolated from the scorpion Buthus martensii
BV2	A mouse-derived microglial cell
EGCG	Epigallocatechin-3-gallate, the principal bioactive constituent of green tea
OPE	Onion peel extract
PKC	Protein kinase C
LPS	Lipopolysaccharide
AA	Arachidonic acid
